# Zerumbone, a cyclic sesquiterpene, exerts antimitotic activity in HeLa cells through tubulin binding and exhibits synergistic activity with vinblastine and paclitaxel

**DOI:** 10.1111/cpr.12558

**Published:** 2018-12-07

**Authors:** Shabeeba M. Ashraf, Jomon Sebastian, Krishnan Rathinasamy

**Affiliations:** ^1^ School of Biotechnology National Institute of Technology Calicut Calicut Kerala India

**Keywords:** anti‐cancer, cell proliferation, combination index, mitotic block, tubulin, zerumbone

## Abstract

**Objectives:**

The aim of this study was to elucidate the antimitotic mechanism of zerumbone and to investigate its effect on the HeLa cells in combination with other mitotic blockers.

**Materials and methods:**

HeLa cells and fluorescence microscopy were used to analyse the effect of zerumbone on cancer cell lines. Cellular internalization of zerumbone was investigated using FITC‐labelled zerumbone. The interaction of zerumbone with tubulin was characterized using fluorescence spectroscopy. The Chou and Talalay equation was used to calculate the combination index.

**Results:**

Zerumbone selectively inhibited the proliferation of HeLa cells with an IC_50_ of 14.2 ± 0.5 μmol/L through enhanced cellular uptake compared to the normal cell line L929. It induced a strong mitotic block with cells exhibiting bipolar spindles at the IC_50_ and monopolar spindles at 30 μmol/L. Docking analysis indicated that tubulin is the principal target of zerumbone. In vitro studies indicated that it bound to goat brain tubulin with a Kd of 4 μmol/L and disrupted the assembly of tubulin into microtubules. Zerumbone and colchicine had partially overlapping binding site on tubulin. Zerumbone synergistically enhanced the anti‐proliferative activity of vinblastine and paclitaxel through augmented mitotic block.

**Conclusion:**

Our data suggest that disruption of microtubule assembly dynamics is one of the mechanisms of the anti‐cancer activity of zerumbone and it can be used in combination therapy targeting cell division.

## 1 INTRODUCTION

Cancer is one of the leading causes of death all over the world, and cancer treatment remains one of the greatest challenges in front of the healthcare professionals.^1^, ^2^ Most of the anti‐cancer agents currently available in the market are non‐selective and could destroy the normal healthy cells. In addition, they could pose greater risk of developing drug resistance in the tumour cells and are ineffective in inhibiting metastasis.^3^ Cancer is a complex disease characterized by an overactive cell cycle that promotes increased cell proliferation and invasion. Hence, chemotherapeutic agents that target cell division are highly valuable in cancer chemotherapy. Mammalian cell division requires the coordinated actions of the cytoskeleton, the membrane proteins, the motor proteins and the cell cycle regulatory proteins which are precisely controlled in space and time.^4^, ^5^ Among the various players in cell division, tubulin is indispensable for mitosis and chromosome segregation, and antimitotic agents targeting tubulin are the most successful in the treatment of various types of tumours.^5^, ^6^ The other essential antimitotic targets include the mitotic kinesins such as Eg5, CENP‐E, MCAK, and MKLP1; the mitotic kinases such as the Aurora family of proteins; the Polo‐like kinase; and the mitotic checkpoint proteins.^5^, ^7^

Microtubules, the key components of the cytoskeleton, are composed of alpha and beta tubulin heterodimer. They are highly dynamic polymers that undergo polymerization and depolymerization in a short span of time and play essential role in the maintenance of cell shape, intracellular trafficking, cell motility and cell signalling apart from cell division and mitosis.^8^ Tubulin has two nucleotide (GTP) binding sites and three well‐characterized drug binding sites such as the colchicine binding site, the paclitaxel binding site and the *Vinca* alkaloid binding site. The GTP binding site is located at the N‐terminal region of the α and the β subunits, and the colchicine binding site is present at the interface of the α‐ β subunit.^8^, ^9^ The paclitaxel binding site is located at the β‐tubulin, and the *Vinca* alkaloids binding site is located in the N‐terminal region of the β‐tubulin subunit close to the GTP binding site.^9^

The clinically successful antitubulin agents such as the paclitaxel and the vinblastine are obtained from plants. Natural product research is gaining a huge attention because many of the phytochemicals exhibit excellent chemopreventive and chemotherapeutic potential in addition to their selectivity against cancer cells and low cost of production.^10^ Natural products such as genistein, apigenin, quercetin, curcumin, berberine, limonene, coumarin, indirubin, brassinin, indole‐3‐carbinol, lycopene and resveratrol are in clinical/preclinical trials either alone or in combination therapy for the treatment of cancer.^11^, ^12^, ^13^ In the present study, we have investigated the anti‐proliferative mechanism of the natural product zerumbone isolated from the plant *Zingiber zerumbet* belonging to the ginger *family *of flowering* plants (Zingiberaceae*). Zerumbone is a sesquiterpene and is reported to exhibit anti‐cancer potential and other pharmacological activities such as anti‐inflammatory, antibacterial, antimalarial and antioxidant properties.^14^, ^15^, ^16^, ^17^ Zerumbone was found to be effective in preventing tumour angiogenesis by inhibiting the VEGF expression and NF‐κB activity.^18^ It was reported to induce apoptosis in various cancer cell lines by modulating the FAS and TRAIL signalling pathways, through enhanced expression of TNF and modulating Bax/Bcl‐2 ratio .^19^, ^20^, ^21^ Recently, zerumbone was reported to block the cell cycle at mitosis^17^, ^22^ and induce apoptosis in cancer cells through inhibition of microtubule assembly.^23^ However, the mechanism behind the mitotic block was not clearly established, and hence, we have performed this study to elaborate its anti‐cancer mechanism through biophysical, biochemical and cell culture studies.

Cell culture studies showed an excellent observation that zerumbone exhibited selective toxicity against HeLa cells through enhanced internalization of the compound and inhibited their migration. The anti‐proliferative effect of zerumbone in HeLa cells correlated well with its ability to inhibit the cell cycle at mitosis through tubulin binding. Zerumbone bound to tubulin at the colchicine binding site and inhibited the polymerization of tubulin into microtubules. Zerumbone exhibited excellent synergistic antimitotic and anti‐proliferative activity in HeLa cells when combined with clinically established drugs such as paclitaxel and vinblastine. Together, the results suggest that the anti‐proliferative effects of zerumbone could be partly through its inhibitory effects on tubulin and induction of mitotic block, and combination of zerumbone and other anti‐cancer drugs might provide a therapeutic advantage in controlling the growth of cancer cells.

## 2 MATERIALS AND METHODS

### 2.1 Materials

Paclitaxel, vinblastine sulphate, podophyllotoxin, colchicine, 5,5′‐dithiobis‐2‐nitrobenzoic acid (DTNB), sulforhodamine B (SRB), Hoechst 33342, guanosine 5′‐triphosphate (GTP), propidium iodide, EGTA, MgCl_2_, piperazine‐*N*,*N*′‐bis (2‐ethanesulphonic acid) (PIPES), mouse monoclonal anti‐α‐tubulin IgG and FITC‐conjugated anti‐mouse IgG, fluorescein isothiocyanate isomer (FITC) and dimethylformamide were purchased from Sigma‐Aldrich (St. Louis, MO, USA). Foetal bovine serum (FBS) and Alexa Fluor 568‐conjugated anti‐mouse IgG were purchased from Invitrogen (Thermo Scientific, Massachusetts, USA). Acridine orange (AO), hydroxysuccinimide, minimal essential medium (MEM), cell culture tested antibiotic solution, and phosphate‐buffered saline (PBS) were purchased from HiMedia (Mumbai, India). Dichloromethane, n‐hexane, hydroxylamine hydrochloride and triethylamine were purchased from Merck, India. 1‐Ethyl‐3‐(3‐dimethylaminopropyl) carbodiimide hydrochloride was purchased from SRL chemicals. All other reagents used in the study were of analytical grade.

### 2.2 Isolation, purification and characterization of zerumbone

Fresh rhizomes of *Zingiber zerumbet* were collected from the farms of the Indian Institute of Spice Research (IISR), Calicut, Kerala (India), and it was authenticated by Dr D Prasath, Principal Scientist, IISR, Calicut. Zerumbone was extracted and isolated from the rhizomes of *Zingiber zerumbet *as described earlier.^24^ Briefly, 1 kg of fresh rhizomes was washed under running tap water and cut into slices. The slices were then shade‐dried at 37°C for 5 days. The dried samples were then soaked in methanol for 3 days, and the methanolic extract was concentrated by using the rotary evaporator (Heidolph Instruments, GmbH & CO. KG, Schwabach, Germany). The extract was then fractionated by silica gel (mesh size 200) column chromatography using organic solution mixture of hexane: ethyl acetate (8:2; v/v). Zerumbone thus obtained was further purified by crystallization. The purity of zerumbone was analysed and confirmed using the Shimadzu liquid chromatography‐mass spectrometry (LC‐MS) and Bruker Avance III Nuclear Magnetic Resonance spectroscopy (NMR) using the standard procedure.

### 2.3 Fluorescent labelling of zerumbone

Zerumbone does not have any characteristic fluorescence; hence, we labelled it with fluorescein isothiocyanate (FITC) by conjugating zerumbone oxime with fluoresceinthiocarbamyl ethylenediamine (EDF) to characterize the binding site of zerumbone on tubulin. Zerumbone oxime was synthesized as described earlier.^25^ In brief, zerumbone (0.3 g) was dissolved in 10 mL of ethanol containing 0.9 g of hydroxylamine hydrochloride and 1.8 g potassium carbonate. The mixture was stirred for 5 hours at room temperature. The reaction mixture was then filtered, and the residue was washed with methanol. The filtrate was concentrated under reduced pressure and was then mixed with dichloromethane (10 mL). The organic layer was collected and washed with water. The resultant mixture was concentrated and dried to get crystalline zerumbone oxime, which was subjected to FTIR analysis. Fluoresceinthiocarbamyl ethylenediamine (EDF) was synthesized as described earlier.^26^ Zerumbone oxime (20 mg), 1‐ethyl‐3‐(3‐dimethylaminopropyl) carbodiimide hydrochloride (20 mg) and hydroxysuccinimide (6 mg) were dissolved in 1 mL of dimethylformamide. The mixture was stirred continuously for 1 hour; and to this mixture, 5 mL of dimethylformamide containing 25 mg of EDF was added dropwise over a period 30 minutes. The reaction mixture was allowed to resolve on a preparative silica gel using ethyl acetate/methanol/acetic acid (90/8/2 v/v/v) as mobile phase. The spots on the preparative TLC plate were identified in a UV chamber, and the spot corresponding to FITC‐conjugated zerumbone (Rf 0.49) was eluted using methanol.^26^

### 2.4 Cell culture and cell proliferation assay

Human cervical cancer cell line (HeLa) and mouse fibroblast cell line (L929) were obtained from the National Centre for Cell Science, Pune, India. The cells were grown in 25 cm^2^ tissue culture flasks in a humidified atmosphere containing 5% CO_2_ and 95% air at 37°C. HeLa cells were grown and maintained in minimal essential medium (MEM) supplemented with 10% (v/v) FBS, sodium bicarbonate and antibiotic solution containing 100 units of penicillin, 100 µg of streptomycin and 0.25 µg of amphotericin B per mL. L929 cells were grown and maintained in Dulbecco's modified Eagle's medium (DMEM) supplemented with 10% FBS, sodium bicarbonate and antibiotic solution. The cytotoxic effect of zerumbone on HeLa cells and L929 cells was determined in 96‐well tissue culture plates using the standard SRB assay.^27^, ^28^

### 2.5 Cellular uptake of fluorescein zerumbone (fluorozerumbone)

HeLa cells and L929 cells (∼1 × 10^6^ cells/mL) were incubated with 0.1% DMSO or 40 µmol/L zerumbone or 40 µmol/L fluorozerumbone for 4 hours. After the incubation period, the cells were collected by trypsinization and counted. Cells were then centrifuged at 800 x *g* for 10 minutes and washed three times with cold PBS. The cell pellet was then dried and suspended in 800 µL of methanol and sonicated till fluorozerumbone is completely extracted into the methanol fraction. The cell lysate was centrifuged at 2000 x *g* for 5 minutes. The absorbance and fluorescence spectra (excitation at 494; emission at 500‐600) of the supernatant containing flourozerumbone were recorded. The total cellular uptake was estimated as mmol/cell.^29^ Standard curve of fluorozerumbone was obtained using the standard solution in the range of 1‐100 µmol/L. Spectral scan was analysed using Systronics AU‐2701 UV‐visible double beam spectrophotometer at 200‐800 nm.

### 2.6 Calculating the percentage of apoptotic cell death using AO staining

HeLa cells (0.5 × 10^5^ cells/mL) grown on poly‐l‐lysine‐coated glass coverslips (12 mm) in 24‐well tissue culture plates were treated with either 0.1% DMSO or different concentrations of zerumbone (10, 20 and 30 μmol/L) for 24 hours. The live cells were immediately viewed under an inverted Nikon ECLIPSE T*i *(Tokyo, Japan) fluorescent microscope after adding AO (2 μg/mL), and the images were captured using the CoolSNAP digital camera.

### 2.7 Cell migration assay

HeLa cells (1 × 10^6^ cells/mL) were grown in minimum essential medium supplemented with 10% FBS in 35 mm cell culture dishes. At 90% confluence, a wound was made using a sterile micropipette tip.^30^, ^31^ The floating cells were removed immediately after wounding, and the media were changed with fresh one containing different concentrations of zerumbone (0, 5, 10 and 15 μmol/L). Cells were observed at 24, 48 and 72 hours of intervals, and the bright‐field images of the wound closure were recorded using the Nikon ECLIPSE T*i *inverted microscope. Percentage wound healing was calculated by using the formula:

$$\%\text{Cell migration}=\left[1-\left(\text{width of scratch at specific time point}\;t/\text{width of the scratch at zero time}\right)\right]\times 100$$

### 2.8 Mitotic index assay and Immunofluorescence microscopy

For determination of mitotic index (MI), HeLa cells were grown on poly‐l‐lysine‐coated glass coverslips (12 mm) in 24‐well tissue culture plates and were subsequently treated with different concentrations of zerumbone for 24 hours. The cells were then fixed with 3.7% (v/v) formaldehyde solution in PBS for 30 minutes at 37°C. The cells were permeabilized with cold methanol at −20°C for 30 minutes. Cells were then stained with Hoechst 33342 (1.5 µg/mL). The coverslips were washed twice with PBS and were mounted on clean glass slides with the mounting medium containing 1,4‐diazabicyclo [2.2.2] octane (DABCO) as anti‐quenching agent. The number of mitotic and interphase cells was counted using a Nikon ECLIPSE T*i*‐E inverted fluorescent microscope (Tokyo, Japan). The MI was calculated as the percentage of cells blocked at mitosis.^31^ At least 1000 cells were counted for each concentrations of zerumbone. The HeLa cells that were treated with different concentrations of zerumbone for 24 hours were fixed with formaldehyde and processed to visualize the interphase and mitotic microtubules using mouse monoclonal alpha‐tubulin antibody and goat anti‐mouse IgG conjugated to Alexa Fluor 568. The DNA was stained with Hoechst 33342 to visualize the DNA. Gamma tubulin staining was performed using rabbit monoclonal anti‐gamma tubulin antibody at 1:1000 dilutions as described earlier.^27^, ^32^, ^33^ Immunofluorescence images were acquired using the CoolSNAP digital camera and were processed by using ImageJ (NIH, USA).

### 2.9 Molecular docking study

The interaction of zerumbone with tubulin dimer and other cell division proteins such as Eg5, Aurora A, Plk1, Kif2 and Nek2 was analysed through molecular docking. The 3D crystal coordinates of tubulin heterodimer (5J2U), Eg5 motor domain (1X88), Aurora A (5LXM), Polo‐box domain of Plk1(4WHK), Kif2 motor domain (2GRY) and Nek2 (2W5A) were obtained from Protein Data Bank (http://www.rcsb.org/pdb/home/home.do). The protein preparation wizard of Glide, Schrodinger Maestro v11.1 (Schrodinger, LLC, New York, NY, USA) was used to prepare the protein structures. The structures were refined by adding missing side chains and removing water molecules, ions, cofactors and inhibitors. Then, they were energy‐minimized until the average RMSD of the non‐hydrogen atoms reached 0.3 Å. The 3D structure of zerumbone was obtained from PubChem (https://pubchem.ncbi.nlm.nih.gov, PubChem CID: 5470187). The low energy conformations of zerumbone were prepared using LigPrep module of Schrodinger. The molecular docking of zerumbone with different proteins was performed using Grid‐Based Ligand Docking with Energetics (Glide) module of Schrodinger. A grid box covering the entire protein was generated to perform blind docking of zerumbone using extra precision (XP) mode of Glide script. The best docking pose was selected based on Glide scoring function, Glide energy, Emodel energy, EvdW and Ecoul using Glide XP Visualizer.

### 2.10 Prime/MM‐GBSA scoring

The binding energy (ΔG binding) of zerumbone to each protein was calculated using Prime/MM‐GBSA method (Schrodinger, LLC) using the equation Δ*G*_bind_ = ΔE_MM_ + Δ*G*_Solv_ + ΔGSA, where ΔE_MM_ is the difference in the minimized energies between protein‐zerumbone complex and the sum of the minimized energies of unbound protein and zerumbone, Δ*G*_Solv_ is the difference in the GBSA solvation energy of protein‐zerumbone complex and the sum of the solvation energies of unbound protein and zerumbone, and ΔGSA is the difference in the surface area energies of complex and the sum of the surface area energies of unbound protein and zerumbone. The Prime/MM‐GBSA calculations were performed based on the protein‐zerumbone complexes obtained from Glide docking using OPLS‐2005 forcefield and VSGB2.0 solvent model.

### 2.11 Purification of tubulin

Goat brain tubulin was isolated by two cycles of polymerization and depolymerization in the presence of glutamate as described earlier.^34^, ^35^ Bradford assay was used to estimate the tubulin concentration using bovine serum albumin as the standard.^36^ The protein was stored in aliquots at −80°C until further use. All the experiments with tubulin were performed in PEM buffer (25 mmol/L PIPES, 1 mmol/L EGTA, 3 mmol/L MgCl_2_, pH 6.8).

### 2.12 Spectral measurements

All the absorbance measurements were carried out in Systronics AU‐2701 UV‐visible double beam spectrophotometer using a cuvette of 1 cm path length. FTIR spectrometer (PerkinElmer, PerkinElmer Inc., Waltham, MA, USA) was used to record the FTIR spectra. The fluorescence measurements are performed in JASCO FP‐8300 spectrofluorometer (Tokyo, Japan) equipped with a thermostatted cell holder directly connected to a circulating water bath for maintaining constant temperature. For all the fluorescence measurements, the inner filter correction was done according to the equation *F* = *F*_obs_ × antilog [(*A*_ex_ + *A*_em_)/2], where *A*_ex_ is the absorbance of ligand at the excitation wavelength, and *A*_em_ is the absorbance of ligand at the emission wavelength.^37^, ^38^ The background fluorescence exhibited by buffer and free ligands was routinely subtracted from all the samples.

### 2.13 Determination of *K*d

For calculating the dissociation constant, tubulin (1 µmol/L) was incubated with varying concentrations of zerumbone in PEM buffer at 37°C for 30 minutes. The samples were excited at 295 nm to specifically excite the tryptophan residues of tubulin, and the emission spectrum was recorded. The fraction of binding sites (X) occupied by zerumbone was evaluated using the equation *X *= (*F*_o_ − *F_c_*)/Δ*F*_max_, where *F_o_* and *F_c_* represent the fluorescence intensity of tubulin in the absence and presence of varying concentrations of zerumbone. The maximum change in the fluorescence intensity, Δ*F*_max_, was calculated from the *Y*‐intercept of the graph 1/Δ*F* vs 1/[zerumbone]. Assuming a single binding site of zerumbone per tubulin dimer, the dissociation constant (*K*d) was estimated using the relationship, 1/*X = *1 + (*K*d/*L*_f_), where *L*_f_ is the concentration of free zerumbone.^30^, ^39^ The experiment was repeated three times.

### 2.14 Sedimentation assay

The in vitro microtubule sedimentation assay was performed to detect the effect of zerumbone on the polymerization of tubulin. Different concentrations of zerumbone were incubated with tubulin (12 μmol/L) in PEM buffer containing 0.8 mol/L glutamate and 1 mmol/L GTP at 37°C for 1 hour. The reaction mixture was then subjected to centrifugation at 50 000 × *g* for 1 hour. The supernatant and pellet were collected separately, and the protein concentration in the supernatant was measured using Bradford assay.^30^

### 2.15 Light scattering assay

The effect of zerumbone on the assembly of microtubule was also analysed by monitoring the kinetics of tubulin polymerization. Different concentrations of zerumbone were added to 12 μmol/L tubulin in the polymerization buffer containing 25 mmol/L PIPES, 1 mmol/L EGTA, 3 mmol/L MgCl2 and 0.8 mol/L glutamate. The assembly reaction was initiated by adding 1 mmol/L GTP and incubated at 37°C.^38^ The polymerization of tubulin was monitored by light scattering at 550 nm for 15 minutes using JASCO FP‐8300 spectrofluorometer (Tokyo, Japan) connected with circulating water bath maintained at 37°C.

### 2.16 Binding site competition assay

Colchicine has a very weak fluorescence in aqueous buffers but exhibits a strong fluorescence after binding to tubulin.^40^ This fluorescence property of colchicine is exploited in binding site competition assays to predict the binding site of unknown compounds. Tubulin (1 μmol/L) was incubated with colchicine (10 μmol/L) for 1 hour at 37°C to form a stable tubulin‐colchicine (T‐C) complex which has several fold higher fluorescence than unbound colchicine.^40^ Different concentrations of zerumbone were then added to the T‐C complex and incubated for further 30 minutes at 37°C. The samples were excited at 360 nm, and the emission spectra were recorded.^30^, ^39^ Alternatively, competition assay was also done using the fluorescence of the tubulin‐fluorozerumbone complex. Tubulin (2 µmol/L) was incubated with 10 µmol/L fluorozerumbone and incubated for 20 minutes at 37°C. After the incubation period, different concentrations of zerumbone (10 µmol/L or 20 µmol/L), or 20 µmol/L colchicine, or 5 µmol/L vinblastine were added to the tubulin‐fluorozerumbone complex and incubated under dark at 37°C for further 30 minutes. The samples were excited at 494 nm, and emission spectra were recorded. The competition assay was repeated using EDF in place of fluorozerumbone with different concentrations of zerumbone (10 and 20 µmol/L) and 20 µmol/L colchicine.

### 2.17 Determination of combination index

HeLa cells were incubated with zerumbone or vinblastine or paclitaxel alone or zerumbone (5, 10 and 12 µmol/L) and paclitaxel (5 and 10 nmol/L) in combination or zerumbone (5, 10 and 12 µmol/L) and vinblastine (6 and 1.2 nmol/L) in combination for 24 hours. The CI was calculated to understand the effect of zerumbone on the cytotoxic activity of paclitaxel and vinblastine. The CI was calculated using the Chou and Talalay^41^ equation:

$$\text{CI}=\left[\left(D\right)1/\left(\mathrm{Dx}\right)1\right]+\left[\left(D\right)2/\left(\mathrm{Dx}\right)2\right]$$

Where, (*D*)1 and (*D*)2 are the concentrations of drug 1 (zerumbone) and drug 2 (vinblastine or taxol) in combination that produces a given effect, (*Dx*)1 and (*Dx*)2 are the concentrations of drug 1 and drug 2 that also produces the same effect when used alone. (*Dx*), the concentration of the drug which produces any particular effect, was calculated from the median effect equation of the Chou and Talalay^41^:

$$\left(\mathrm{Dx}\right)=D_{m}{\left[f_{a}/\;f_{u}\right]}^{1/m}$$

where *D_m_* is the median dose, *f_a_* is the fraction affected, and *f_u_* is the fraction unaffected (*f_u_* = 1 − *f_a_*). The median dose (*D_m_*) was calculated as described earlier.^42^ A CI < 1 indicates synergism, CI = 1 shows additivity, and CI > 1 specifies antagonism. HeLa cells grown on coverslips in 24‐well tissue culture plate were treated with zerumbone in combination with paclitaxel or vinblastine and processed to visualize microtubules and DNA.

## 3 RESULTS

### 3.1 Isolation and characterization

**Figure 1:**
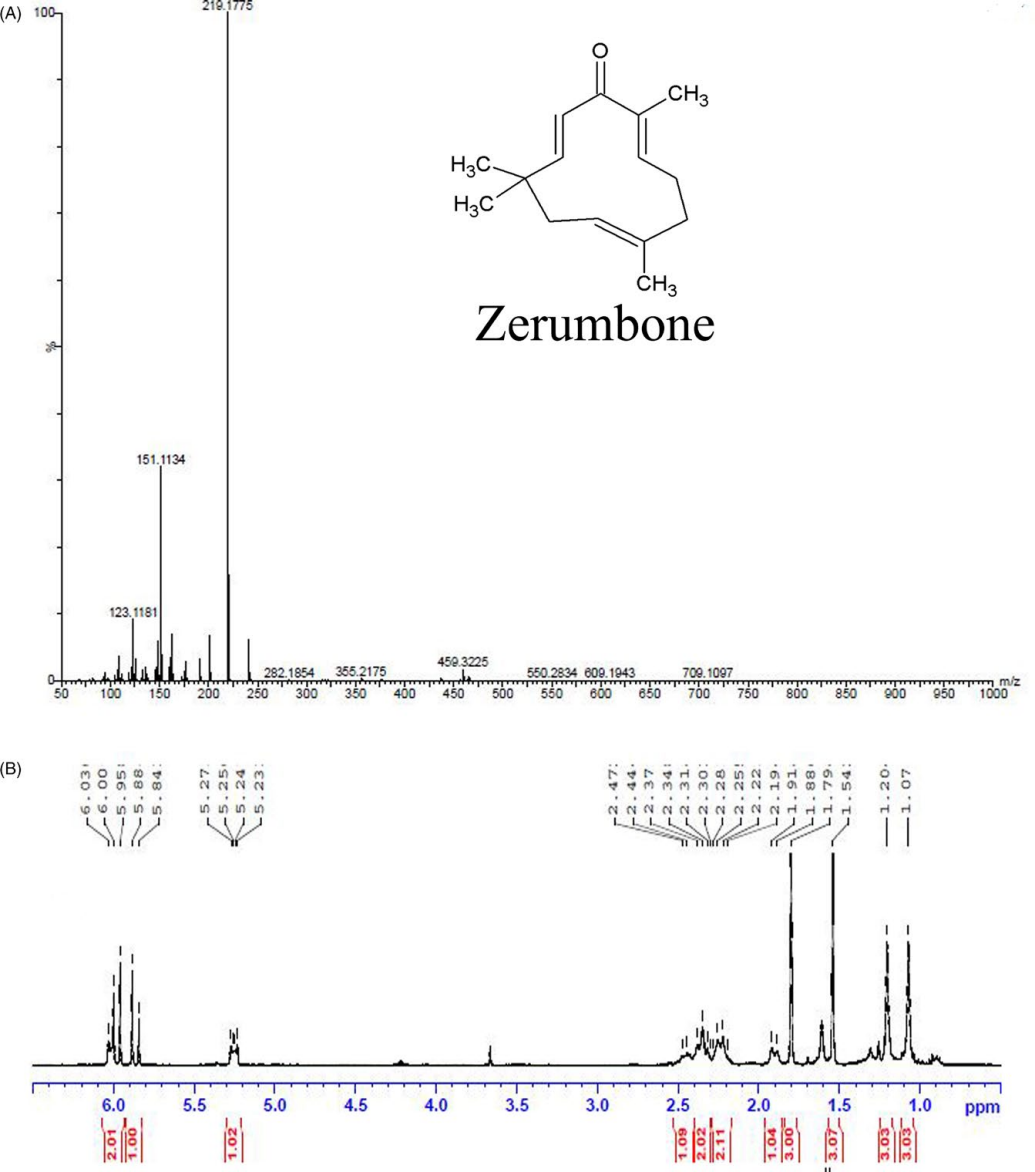
Characterization of zerumbone isolated from *Zingiber zerumbet.* A, LC‐MS analysis of zerumbone. Zerumbone exhibited an M+ ion at 219.17. The inset shows the chemical structure of zerumbone [(2E,6E,10E)‐2,6,9,9‐tetramethylcycloundeca‐2,6,10‐trien‐1‐one]. B, ^1^H‐NMR spectrum of zerumbone (500 MHz, CDCl3)

The methanolic extract of 1 kg of fresh *Zingiber zerumbet* extract yielded 1.8 g of crystalline zerumbone. The isolated product showed M+ ion at 219 when analysed by LC‐MS (Figure 1A). The compound was further analysed and confirmed by ^1^H‐NMR (500 MHz, CDCl3) δ = 6‐6.03 (1H,d), 5.95 (1H,s), 5.88‐5.84(1H,s), 5.23‐5.27(1H,m), 2.47‐2.28 (3H,m), 2.19‐2.25(2H,d), 1.88‐1.91(3H,d), 1.54 (3H, s), 1.79(3H, s), 1.2(3H,s), 1.07(3H,s) (Figure 1B).

### 3.2 Selective toxicity of zerumbone on the proliferation of cancer cells

**Figure 2:**
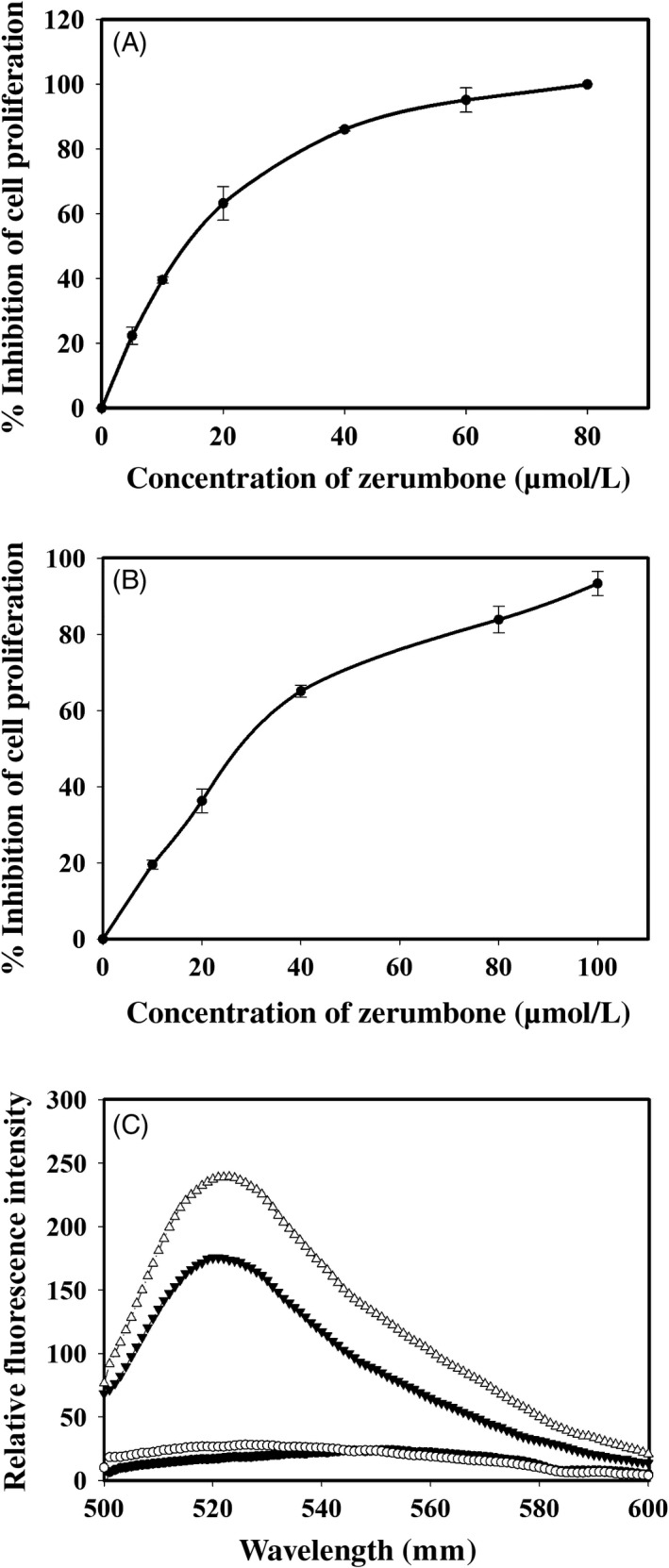
Zerumbone differentially inhibited the proliferation of HeLa (A) and L929 (B) cells. The inhibition of proliferation was determined by the standard SRB assay after treating the cells with different concentration of zerumbone. C, Fluorescence spectra of fluorozerumbone extracted from (▲) L929 and (∆) HeLa cells treated with 40 µmol/L fluorozerumbone. Control L929 (●) and HeLa (○) cells were treated with 0.1% DMSO

Zerumbone inhibited the proliferation of human cervical cancer cell line (HeLa) in a concentration‐dependent manner. After 24 hours of incubation, the half‐maximal inhibitory concentration (IC_50_) of zerumbone on HeLa cells was found to be 14.2 ± 0.5 μmol/L (Figure 2A). Cells treated with 20, 40, 60 and 80 μmol/L zerumbone inhibited the cell proliferation by 63%, 86%, 98% and 100%, respectively. We analysed the effect of zerumbone on L929 cells since it is generally used as an ideal in vitro model to test the chemical toxicity, drug cytotoxicity and material biocompatibility.^43^, ^44^ Interestingly, zerumbone exhibited less cytotoxic effect on normal mouse fibroblast cells (L929) with an IC_50_ of 30.5 ± 1.5 μmol/L (Figure 2B). Cells treated with 10, 20, 40 and 80 μmol/L zerumbone inhibited the growth of L929 cells by 20%, 33%, 70% and 83%, respectively.

### 3.3 Internalization of fluorozerumbone by HeLa and L929 cells

The fluorozerumbone internalized by HeLa and L929 cells treated with 40 µmol/L fluorozerumbone for 4 hours was extracted using methanol and quantified based on the absorption spectra of the standard fluorozerumbone. We found that the uptake of fluorozerumbone by HeLa cells was 26 nmol/cell and that by L929 cells was 14.8 nmol/cell. The methanolic extracts were further subjected to fluorometric analysis by exciting them at 494 nm as explained in *Materials and methods*. As shown in Figure 2C, the fluorescence intensity of the extract obtained from HeLa cells was 16% higher than that of the extract obtained from L929 cells. These results indicate that the cellular uptake of fluorozerumbone is much higher in tumour cells.

### 3.4 Zerumbone‐induced apoptosis in HeLa cells

**Figure 3:**
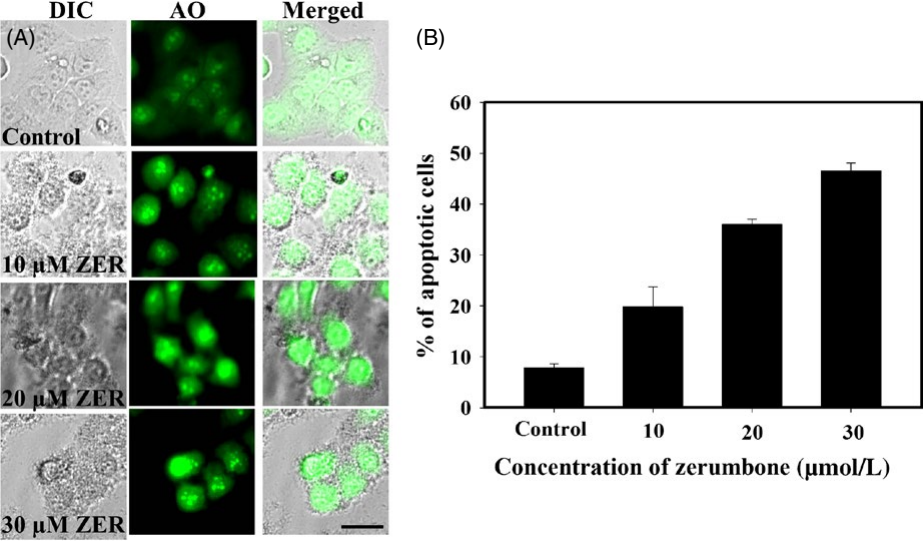
Zerumbone‐induced apoptosis in HeLa cells in a dose‐dependent manner. A, HeLa cells (0.5 × 10^5^ cells/mL) were incubated with different concentrations of the zerumbone for 24 h. After the incubation period, AO was added and the live cells were viewed under the fluorescent microscope using FITC filter. Apoptotic cells appeared brightly stained with hypercondensed nucleus. Scale bar represents 20 μm. B, Graph represents percentage of apoptotic cells observed after 24 h of treatment with different concentrations of zerumbone. At least, 600 cells were counted for each concentration. The experiment was repeated thrice, and data represent mean ± SD

Acridine orange staining is a common method used to detect apoptotic cell death. After 24 hours, the control cells remained viable and healthy and the zerumbone‐treated cells displayed brightly stained hypercondensed nucleus and membrane blebbing, which indicated the characteristic of apoptosis (Figure 3A). Approximately 20% and 36% apoptotic cells were detected in the cells treated with 10 and 20 μmol/L zerumbone for 24 hours (Figure 3B). At higher concentration of zerumbone (30 μmol/L), ~50% cells were found to be in the later stages of apoptotic cell death with the characteristic features such as membrane blebbing and cell shrinkage. Under similar conditions, the number of apoptotic cells in the control was 8%.

### 3.5 Zerumbone inhibited the migration of HeLa cells in a concentration‐dependent manner

**Figure 4:**
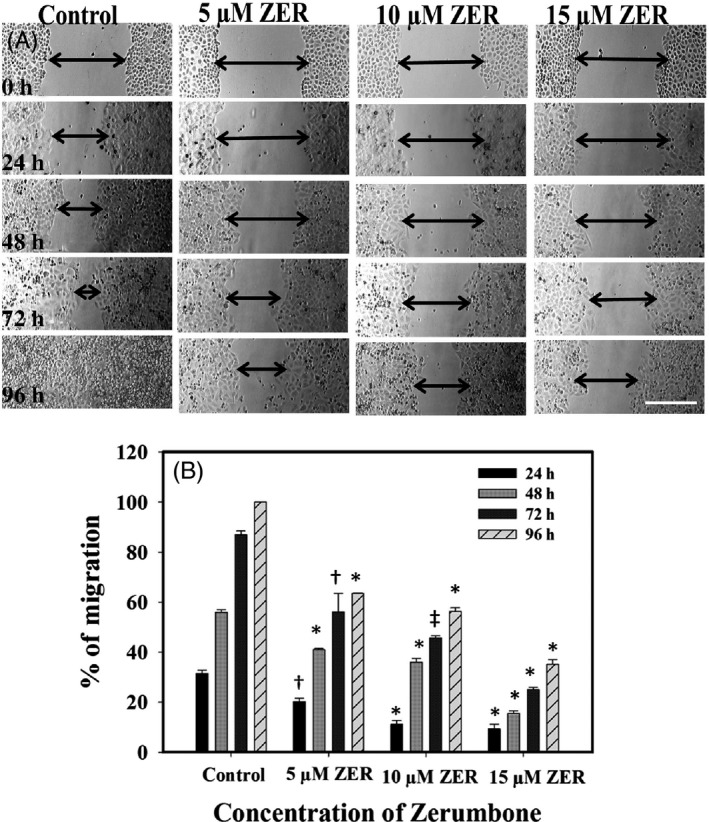
Effect of zerumbone on the migration of HeLa cells. A, Migration of HeLa cells in the absence and presence of the indicated concentrations of zerumbone at different time intervals (24, 48 72 and 96 h) was monitored as mentioned in Section 2. The scale bar represents 200 µm. B, Percentage of cell migration at specific time intervals in the absence and presence of 5, 10 and 15 µmol/L zerumbone was calculated as described in Section 2. The data shown are mean ± SD of three independent experiments (*P < 0.001; ‡ *P *< 0.01; †*P < *0.05)

Wound healing assay was used to check the migration of HeLa cells upon treatment with different concentrations of zerumbone. As shown in Figure 4A, zerumbone effectively inhibited the migration of cancer cells even at concentrations lower than the IC_50_. After 24 hours, the control cells have shown 30% migration; during the same time, 5, 10 and 15 μmol/L zerumbone exhibited 20%, 11% and 9% migration, respectively. After 48 hours, the control cells displayed ~56% wound healing, and in the cells treated with zerumbone 5, 10 and 15 μmol/L, the wound healing was found to be 41%, 36% and 15%, respectively. Similar concentration‐dependent inhibition of wound healing was observed after 72 hours of treatment, and the complete wound healing was observed in control cells after 96 hours, while 63%, 56% and 35% wound healing were observed in the cells treated with 5, 10 and 15 μmol/L zerumbone (Figure 4B). The results suggest that zerumbone can effectively prevent the migration of cancer cells in a concentration‐ and time‐dependent manner even at concentrations lower than the IC_50_.

### 3.6 Zerumbone caused depolymerization of interphase and mitotic microtubules in HeLa cells and blocked the cells at mitosis

**Figure 5:**
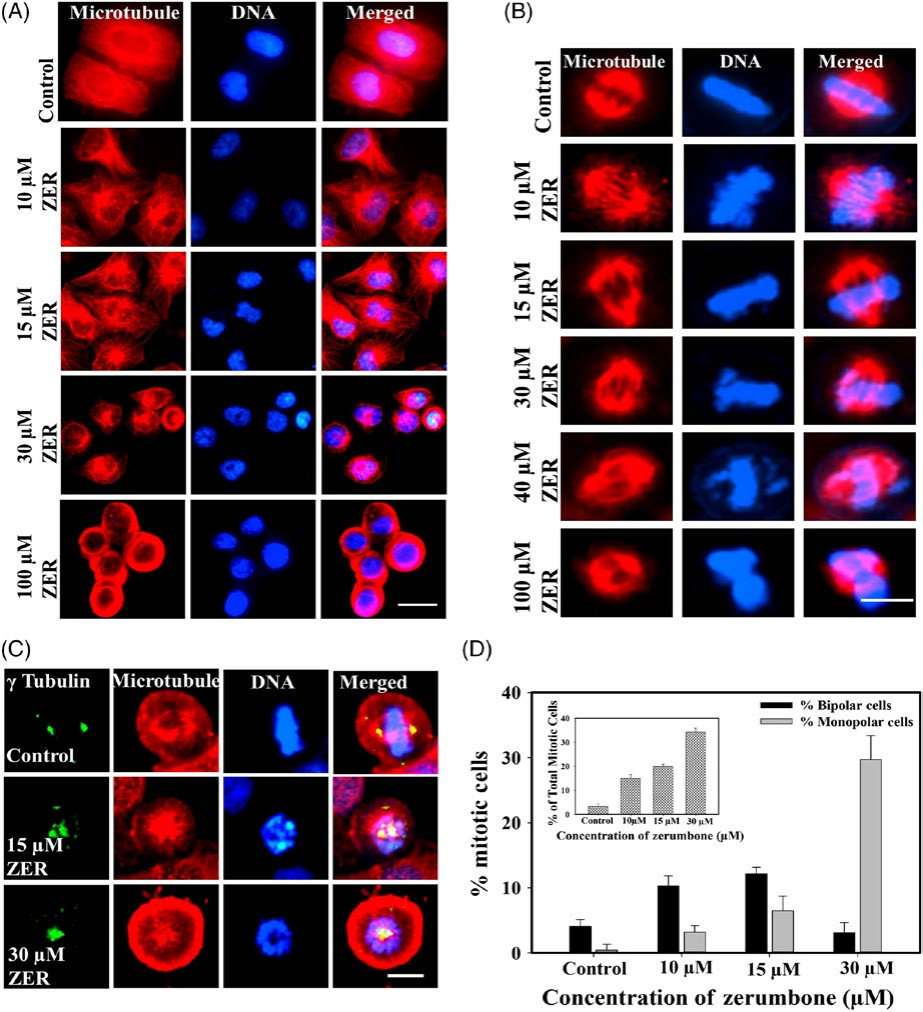
Effect of different concentrations of zerumbone on interphase and mitotic microtubules in HeLa cells. A, HeLa cells were incubated with the indicated concentrations of the zerumbone for 24 h. Microtubules (red) and DNA (blue) were visualized as mentioned in Section 2. Scale bar represents 20 μm. B, Effect of zerumbone on the spindle microtubules and chromosome organization in HeLa cells. Zerumbone induced abnormal spindles and misalignment of chromosomes at the metaphase plate. Scale bar represents 10 μm. C, Zerumbone induced the formation of monopolar spindles in HeLa cells. HeLa cells were incubated with different concentrations of zerumbone for 24 h, and the cells were then fixed and processed to visualize centrosomes, DNA and microtubules. Scale bar represents 5 μm. D, Zerumbone treatment increased the number of mitotic cells with monopolar spindles in HeLa cells. Percentage of cells with bipolar (black) and monopolar (grey) spindles post‐treatment with zerumbone for 24 h are shown in the graph. Inset shows the percentage of total mitotic cells. All the experiments were performed three times. The data represent mean ± SD

Zerumbone induced significant depolymerization of interphase and mitotic microtubules in HeLa cells. The control cells treated with vehicle (0.1% DMSO) exhibited typical microtubule organization with the microtubules spread over the entire cell. Zerumbone at the IC_50_ (15 μmol/L) and lower concentrations such as 10 μmol/L did not alter the interphase microtubule network (Figure 5A). However, higher concentrations such as 30 and 100 μmol/L caused significant disruption of the interphase microtubules with shrunken cells. Similarly, zerumbone did not cause significant disruption of the mitotic spindles at the IC_50_ (15 μmol/L) and lower concentrations but perturbed the organization of the chromosomes at the metaphase plate (Figure 5B). Surprisingly, we found that significant number of the mitotic cells had monopolar spindles. Hence, the mitotic cells were further analysed by observing the centrosome using gamma tubulin staining. As shown in Figure 5C, zerumbone inhibited the centrosome separation and caused the formation of monopolar spindles with rosette‐like chromosomes around it. The estimation of HeLa cells blocked at mitosis 24 hours of post‐treatment with zerumbone revealed a strong mitotic block with both bipolar and monopolar spindles. In the cells treated with 10, 15, and 30 μmol/L zerumbone, the number of cells with monopolar spindles was calculated to be 3%, 7% and 27%, respectively, and the MI, which is the ratio of the total number of the mitotic cells to the total cells, was found to be 15%, 20% and 34%, respectively. Under similar conditions, the MI of the control cells was 3.5% (Figure 5D).

### 3.7 Probing the possible targets of zerumbone through computational docking analysis

**Figure 6:**
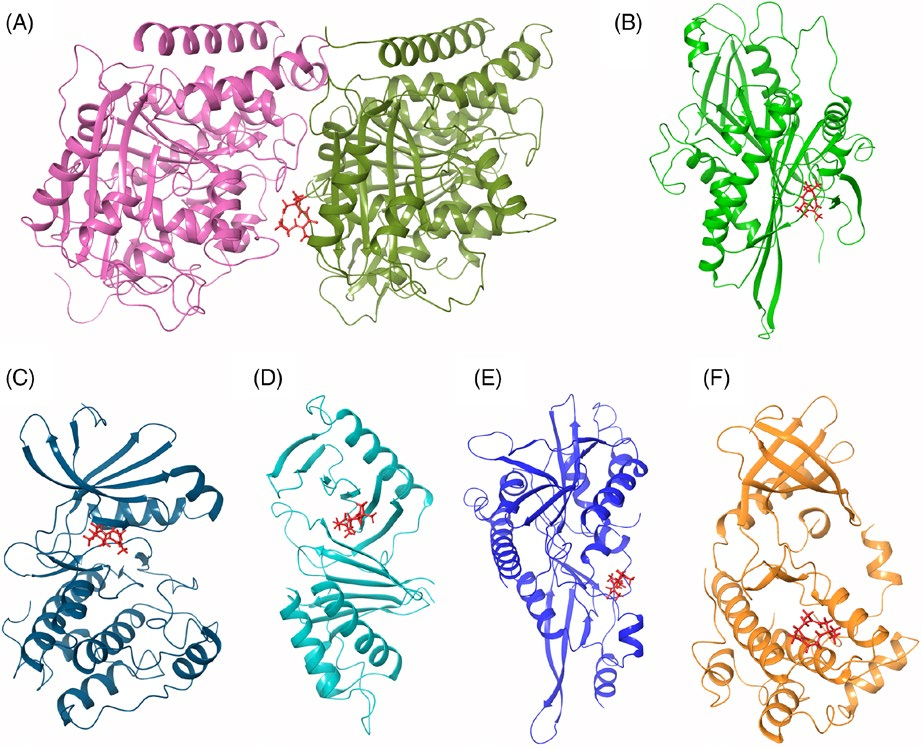
Computational docking analysis of zerumbone with different cell division proteins. Interaction of zerumbone with A, tubulin heterodimer (5J2U), B, Eg5 motor domain (1X88), C, Aurora A (5LXM), D, Polo‐box domain of Plk1(4WHK), E, Kif2 motor domain (2GRY) and F, Nek2 (2W5A)

Since zerumbone treatment produced cells with mitotic abnormalities, we investigated the interaction of the zerumbone with cell division proteins such as tubulin, Eg5, Aurora A, Plk1, Kif2 and Nek2 that play role in the organization of mitotic spindle and organization of chromosomes at the metaphase plate through computational docking. Results of the docking analysis with tubulin implied that zerumbone bound at the interface of the α/β‐tubulin dimer with a Glide docking score of −3.608 kcal/mol (Figure 6). The MM‐GBSA scoring has shown that zerumbone has bound to tubulin dimer at this position with a strong affinity (Δ*G* = −50.638 kcal/mol). The number of hydrogen bonds and the list of protein residues interacting with zerumbone are given in the Table 1. The interaction between tubulin dimer and zerumbone was stabilized by one hydrogen bond with Valβ355 and many hydrophobic interactions with residues Valα177, Proα222, Tyrα224, Leuβ248 and Metβ325. Comparative analysis of the binding sites of the ligands such as paclitaxel,^45^ colchicine^46^ and vinblastine^47^ on the tubulin heterodimer gave an inference that zerumbone binding site partially overlaps with the DAMA colchicine binding site. The residues that both zerumbone and DAMA colchicine interact in the tubulin heterodimer are Serα178, Thrα179, Leuβ248 and Alaβ354 (Table 1). The docking results of other cell division proteins such as Eg5, Aurora kinase A, Polo‐like kinase 1 (Plk1), Kif2A and NIMA‐related kinase 2 (Nek2) are shown in Figure 6B‐F. Based on the Glide docking score (Table 1) and MM‐GBSA scoring, it is possible to speculate that Eg5 and Aurora kinase A could also be the potential target for zerumbone in addition to tubulin.

**Table 1:** Computational docking analysis of zerumbone with tubulin, Eg5 motor domain, Aurora A, Polo‐box domain of Plk1, Kif2 motor domain and Nek2

Protein	Docking score (kcal/mol)	Δ*G* binding (kcal/mol)	No. of H‐ bonds	Interacting residues
Tubulin heterodimer	−3.608	−50.638	1 (Valβ355)	Glnα176, Valα177, Serα178, Thrα179, Argα221, Proα222, Thrα223, Tyrα224, Glnβ247, Leuβ248, Metβ325, Thrβ353, Alaβ354, Valβ355
Eg5	−4.672	−42.675	1 (Val194)	Ser159, Leu161, Asp187, Arg189, Asn190, Gly193, Val194, Ile195, Ile196, Leu199, Glu201, Val238, Ser240, Thr242, Lys260, Asn262, Ile319
Aurora A	−3.657	−50.326	‐	Leu139, Gly140, Lys141, Lys143, Val147, Lys162, Leu210, Gly216, Thr217, Arg220, Glu260, Asn261, Leu263, Ala273, Asp274
Plk1	−2.636	−30.562	1 (Trp514)	Ser418, Leu435, Phe436, Asn437, Ser439, Thr513, Trp514
Kif2a	−2.590	−30.574	1 (Asn207)	Arg204, Pro205, Asn207, Gly289, Ser290, Gly291, Hie294
Nek2	−2.445	−38.151	‐	Val97, Lys100, Gly101, Glu104, Gln106, Tyr107, Leu108, Asp109, Phe112

Glide docking score, Δ*G* binding, number of hydrogen bonds and interacting residues are shown.

### 3.8 Binding of zerumbone to tubulin

**Figure 7:**
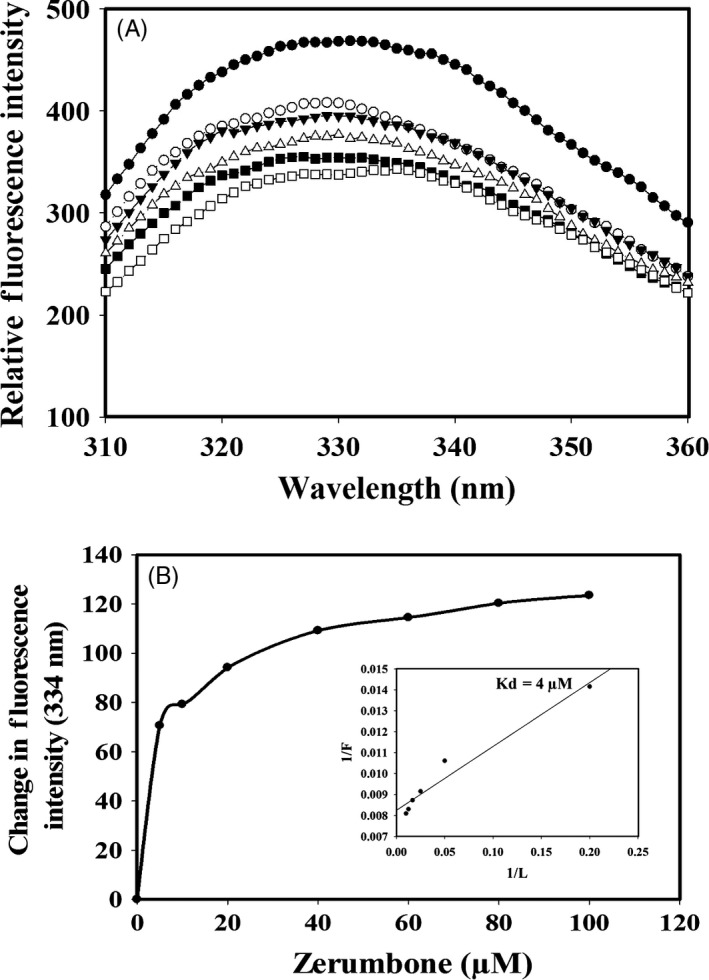
Zerumbone bound to tubulin and quenched the intrinsic tryptophan fluorescence in a concentration‐dependent manner. A, Tubulin (1 µmol/L) was incubated with zerumbone 0 (●), 5 (○), 10 (▼), 20 (∆), 40 (■) and 80 (□) µmol/L for 30 min at 37°C. The samples were then excited at 295 nm, and the emission spectrum was recorded. B, The change in the intrinsic tryptophan fluorescence intensity was plotted against different concentrations of zerumbone. Inset shows the double reciprocal plot, which yielded a Kd of 4 μmol/L

Results from the cell culture studies and docking analysis indicated that tubulin could be one of the primary targets for Zerumbone. Hence, binding of zerumbone on tubulin was analysed using spectrofluorometer by measuring the intrinsic tryptophan fluorescence of tubulin. Zerumbone quenched the intrinsic fluorescence of tubulin in a concentration manner (Figure 7A). Figure 7B shows the change in the fluorescence intensity of tubulin incubated with different concentrations of zerumbone and the analysis of the reduction in the fluorescence of tubulin as a function of zerumbone concentration yielded a dissociation constant (Kd) of 4 μmol/L (Figure 7B inset).

### 3.9 Zerumbone inhibited the polymerization of tubulin in vitro

**Figure 8:**
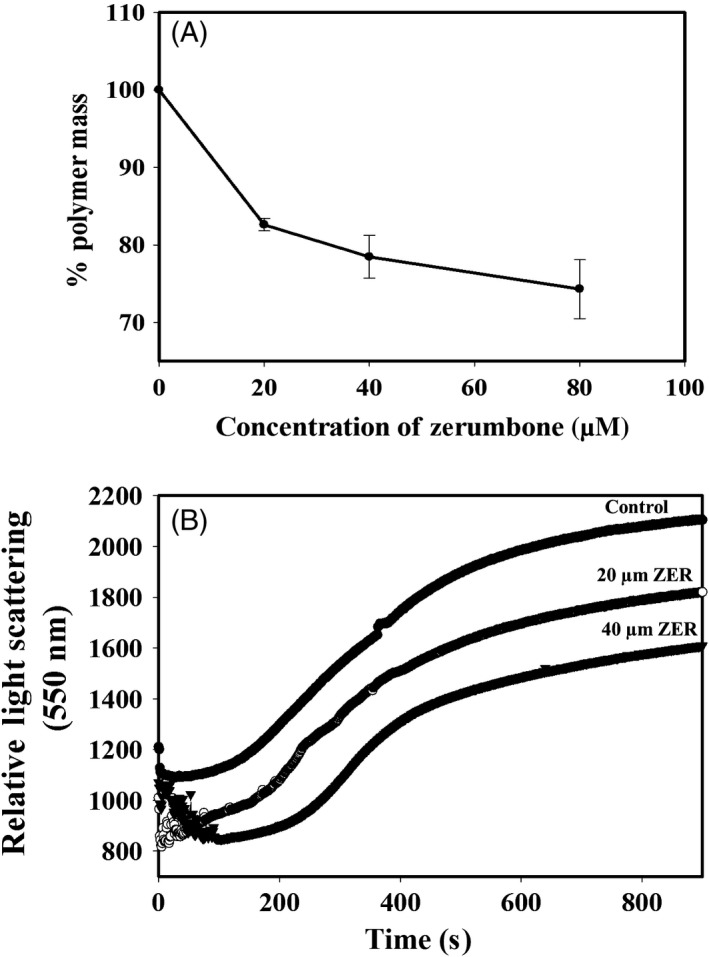
Effect zerumbone on the assembly of tubulin. A, A sedimentation assay was carried out with different concentrations of zerumbone (20, 40 and 80 µmol/L) to determine the percentage of polymer mass of tubulin. The experiment was done three times, and the data represent mean ± SD. B, Zerumbone inhibited the glutamate‐induced polymerization of tubulin. The assembly kinetics of tubulin in the presence and absence of zerumbone 0 (●), 20 (○) and 40 (▼) µmol/L was monitored by recording the light scattering at 550 nm for 15 min as described in Section 2

The effect of zerumbone on tubulin assembly was analysed by using the sedimentation assay and the light scattering assay. Tubulin (12 µmol/L) was allowed to polymerize in the presence or absence of different concentrations of zerumbone as explained in *Materials and methods.* Zerumbone inhibited the polymerization of tubulin in a concentration‐dependent manner (Figure 8A). The polymer mass of tubulin treated with 20, 40 and 80 µmol/L zerumbone was found to be 82.6%, 78.5% and 74.3%, respectively, compared to the control, which is considered as 100%. The kinetics of tubulin polymerization upon treatment with zerumbone was analysed using light scattering assay. Similar to the results obtained in the sedimentation assay, light scattering assay also indicated inhibition of tubulin polymerization by zerumbone (Figure 8B), in a concentration‐dependent manner. At 20 μmol/L zerumbone, the polymer mass was inhibited by 12%, and at 40 μmol/L, the polymer mass was decreased by 20% compared to control. The steady‐state reading at the saturation point of polymerization was taken for the calculation of inhibition of polymerization.

### 3.10 Zerumbone competes with colchicine for the binding site on tubulin

**Figure 9:**
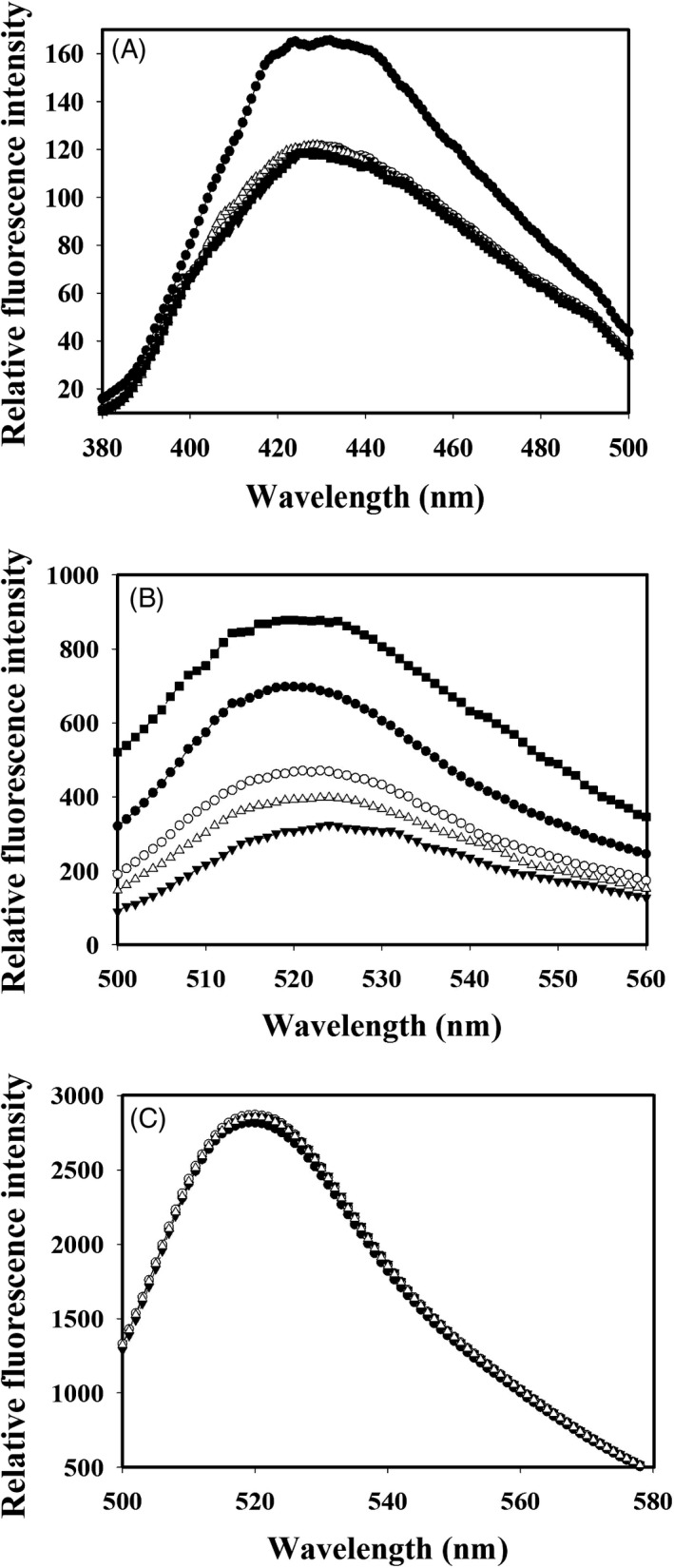
Competition experiment of zerumbone and fluorozerumbone with colchicine, podophyllotoxin and vinblastine for the localization of binding site on tubulin. A, Colchicine (10 μmol/L) was allowed to form complex with tubulin (2 μmol/L) for 1 h at 37°C. Different concentrations of zerumbone 0 (●), 5 (○), 10 (▼) and 20 (∆) µmol/L were added to the complex. Podophyllotoxin 40 (■) µmol/L was used as a positive control. The samples were excited at 360 nm, and the emission spectrum was recorded. B, Tubulin (2 μmol/L) was incubated with 10 μmol/L flourozerumbone for 20 min at 37°C. This was followed by the addition of 0 (●), 10 (○) and 20 (▼) µmol/L zerumbone to the tubulin‐fluorozerumbone complex. Colchicine 20 (∆) µmol/L and vinblastine 5 (■) µmol/L were used as positive controls. All the samples were excited at 494 nm, and emission spectra were recorded. (C) The competition assay was repeated using EDF in place of fluorozerumbone with different concentrations of zerumbone 0 (●), 10 (○) 20 (▼) and 20 (∆) µmol/L colchicine

The effect of zerumbone on the binding of colchicine to tubulin was analysed by monitoring the T‐C fluorescence in the presence of increasing concentrations of zerumbone. As shown in Figure 9A, zerumbone quenched the T‐C fluorescence in a concentration‐dependent manner. Zerumbone at 5 µmol/L quenched the T‐C fluorescence by 29%, and it is clear from the figure that at concentrations more than 5 µmol/L zerumbone, the fluorescence quenching was nearly equal to 30%. This suggests that 5 µmol/L zerumbone is the saturating concentration. Under similar conditions, 40 µmol/L podophyllotoxin, which is reported to bind on the colchicine site of tubulin, quenched the fluorescence of T‐C complex by 32%. To further confirm the binding site of zerumbone on tubulin, we have carried out the competition assay using fluorozerumbone, zerumbone and colchicine by analysing the fluorescence exhibited by fluorozerumbone. As shown in Figure 9B, zerumbone 10 µmol/L quenched the fluorescence of fluorozerumbone by 33%. Approximately 60% quenching of fluorescence was observed with 20 µmol/L zerumbone, indicating that fluorozerumbone binds to the zerumbone binding site and labelling with FITC has not altered its binding site. Addition of 20 µmol/L colchicine to the tubulin‐fluorozerumbone reduced the fluorescence by 44%, confirming that colchicine and zerumbone might share their binding site. Since zerumbone induced depolymerization of microtubules, we also analysed its competition with vinblastine and found that vinblastine (5 µmol/L) enhanced the fluorescence of fluorozerumbone by 44%. The results suggest that binding of vinblastine to tubulin might stabilize the tubulin‐fluorozerumbone complex and both the ligands could bind simultaneously to tubulin. To confirm that the observed effects are not due to the fluorescence tag (EDF), we repeated the same competition assay with EDF in place of fluorozerumbone and found that both zerumbone and colchicine could not reduce the EDF fluorescence (Figure 9C).

### 3.11 Zerumbone inhibited the proliferation of HeLa cells synergistically in combination with vinblastine and paclitaxel

**Figure 10:**
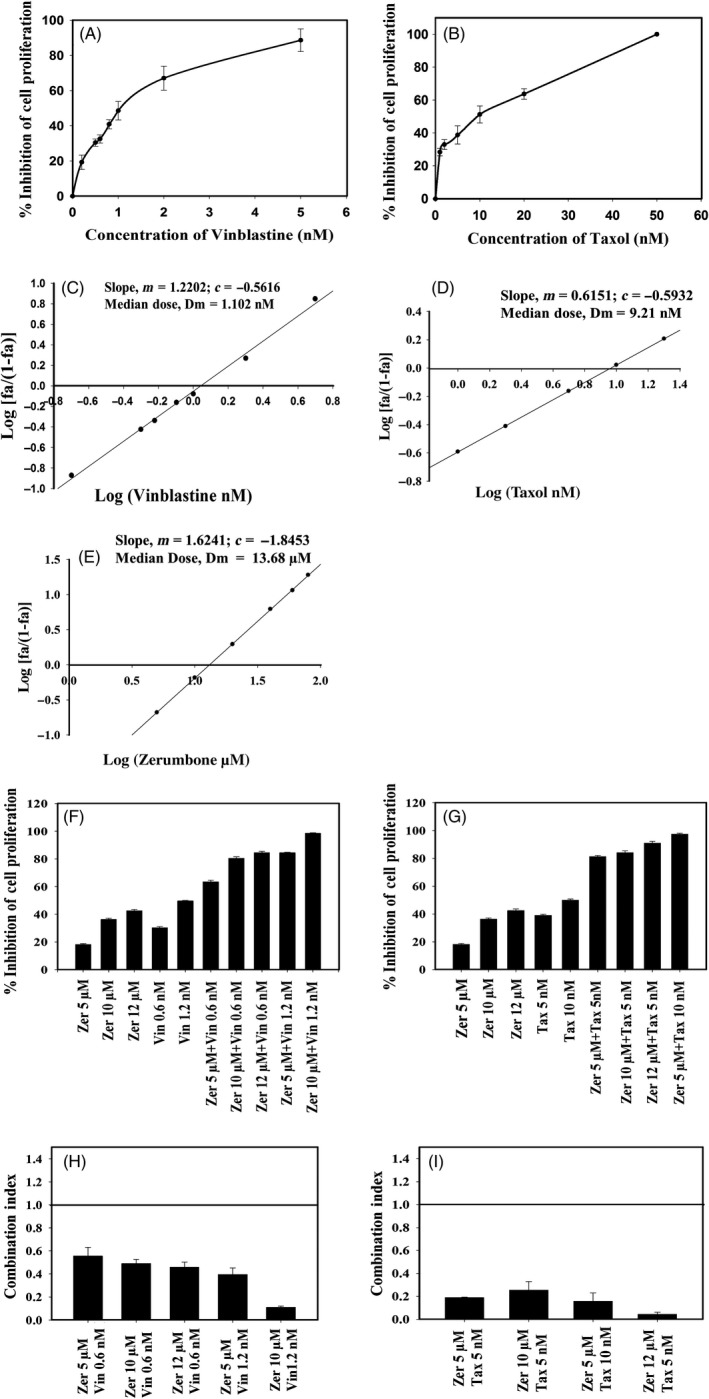
Zerumbone inhibited the proliferation of HeLa cells in synergism with vinblastine and paclitaxel. HeLa cells were treated with different concentrations of vinblastine (A) and paclitaxel (B) for 24 h, and the inhibition of cell proliferation was determined using SRB assay as described in Section 2. Median effect plot for the inhibition of cell proliferation by vinblastine (C), paclitaxel (D) and zerumbone (E). Zerumbone synergistically inhibited the proliferation of HeLa cells in combination with vinblastine (F) and paclitaxel (G). The combined effect of zerumbone with vinblastine (H) and paclitaxel (I) on the proliferation of HeLa cells was calculated using the Chou and Talalay equation, and the CI at different drug concentrations was calculated. All the experiments were performed three independent times, and the error bars represent mean ± SD

Vinblastine and paclitaxel are FDA‐approved clinically used drugs for the treatment of various types of tumours.^48^ The effect of zerumbone was similar to other classical antimitotic drugs; hence, to further explore its potential cancer therapeutics we performed the combination studies with vinblastine and paclitaxel. Vinblastine and paclitaxel inhibited the HeLa cell proliferation with an IC_50_ of 1.2 and 10 nmol/L and a median dose of 1.10  and 9.21 nmol/L, respectively (Figure 10A‐D). The logarithmic plot of the cytotoxic data yielded a median dose of 13.68 µmol/L for zerumbone (Figure 10E). Zerumbone synergistically inhibited the proliferation of HeLa cells when combined with vinblastine and paclitaxel. When 0.6 nmol/L vinblastine was combined with zerumbone of 5, 10 and 12 µmol/L, the inhibition of proliferation of HeLa cell was determined to be 65%, 82% and 86%, respectively. When 5, 10 and 12 µmol/L zerumbone was combined with 1.2 nmol/L vinblastine, the inhibition of proliferation was found to be 84%, 98% and 100%, respectively (Figure 10F). The combination index was calculated based on the Chou and Talalay equation as explained in Materials and methods to demonstrate quantitatively the relationship between the combination of zerumbone with vinblastine and paclitaxel. The CI for the combination of 0.6 nmol/L vinblastine and 5, 10 and 12 µmol/L zerumbone was calculated to be 0.55, 0.48 and 0.45, respectively (Figure 10G) and CI for the combination of 1.2 nmol/L vinblastine with 5 and 10 µmol/L zerumbone was found to be 0.39 and 0.10, respectively (Figure 10G). Combination of 5 nmol/L paclitaxel with 5, 10 and 12 µmol/L of zerumbone inhibited the proliferation of HeLa cells by 82%, 85% and 92%, respectively (Figure 10H). When paclitaxel 10 nmol/L was combined with 5 µmol/L zerumbone, the inhibition of proliferation of HeLa cell was found to be 98%. The CI of 5, 10, and 12 µmol/L zerumbone with 5 nmol/L paclitaxel was calculated to be 0.18, 0.25 and 0.15, respectively. When 5 µmol/L zerumbone was combined with 10 nmol/L paclitaxel, the CI was calculated to be 0.04 (Figure 10I). All the calculated combination indices were found to be lesser than 1, signifying that the combination of zerumbone‐vinblastine and zerumbone‐paclitaxel is strongly synergistic in inhibiting the proliferation of HeLa cells.

**Figure 11:**
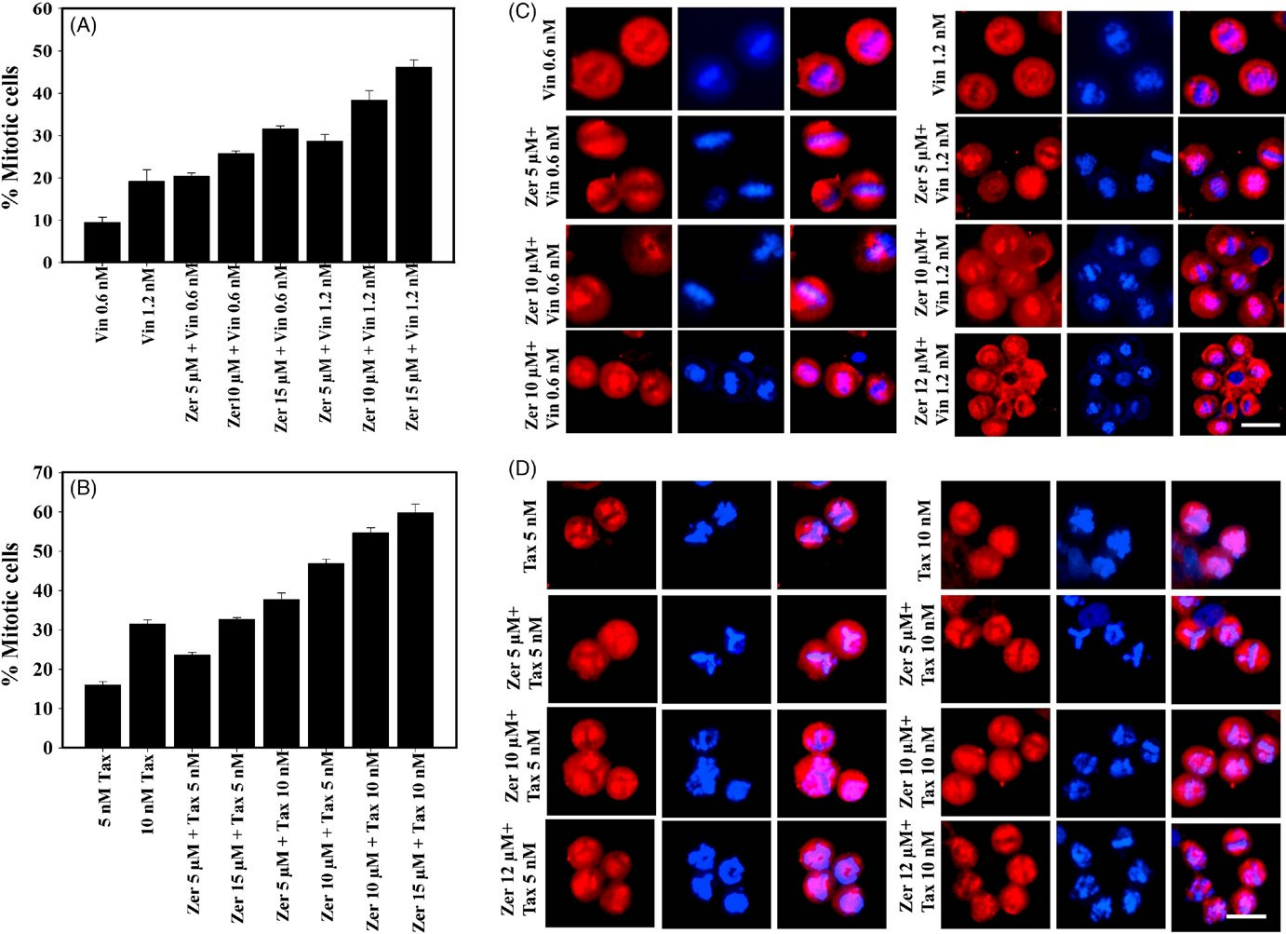
Zerumbone potentiated the mitotic block in HeLa cells in combination with vinblastine and paclitaxel. Zerumbone increased the number of mitotic cells in HeLa cells in combination with vinblastine (A) and paclitaxel (B). All the experiments were performed three times. The data represents mean ± SD. Effect of zerumbone on the spindle microtubule and chromosome alignment in combination with vinblastine (C) and paclitaxel (D). HeLa cells treated with indicated concentrations of zerumbone, vinblastine and paclitaxel were fixed and processed for immunofluorescence microscopy as described in Section 2. The scale bar represents 10 μm

The effect of the combination of zerumbone with vinblastine and paclitaxel on mitotic cells was analysed by calculating the MI. Zerumbone synergistically increased the MI in combination with vinblastine and paclitaxel. As shown in Figure 11A, vinblastine when used alone induced a mitotic block of 9.4%; however, when combined with zerumbone 5, 10 and 12 µmol/L, the mitotic block was increased to 20%, 25% and 31%, respectively. Similarly, vinblastine 1.2 nmol/L when used alone induced a mitotic block of 19%, and when combined with zerumbone 5, 10 and 12 µmol/L, the mitotic block was found to be increased 28%, 38% and 46%, respectively. Zerumbone induced a significant hike in the mitotic cells when combined with paclitaxel, similar to its synergistic activity with vinblastine. When 5 nmol/L paclitaxel was combined with 10 and 12 µmol/L zerumbone, the MI was found to be 32% and 37%, respectively (Figure 11B), and when 10 nmol/L paclitaxel was combined with 10 and 12 µmol/L zerumbone, the MI was increased to 54% and 60%, respectively, while paclitaxel alone at 5 and 10 nmol/L induced 15% and 31% mitotic block (Figure 11B). In addition to the enhanced mitotic arrest, the combined addition of two drugs induced drastic mitotic abnormalities in the organization of the mitotic spindle and alignment of chromosomes (Figures 11C,D).

## 4 DISCUSSION

Several secondary metabolites from plants have successful application as chemotherapeutic agents either in their unmodified form such as paclitaxel, vinblastine, vincristine, camptothecin and podophyllotoxin or in the synthetically modified form such as docetaxel, vinorelbine, vinflunine, topotecan and etoposide.^49^, ^50^ The significance of plants as a major source of anti‐cancer agents can be understood from the fact that most of the presently used chemotherapeutic agents are derived from natural sources in one way or the other.^51^ In this study, we have found that the potential mechanism behind the anti‐cancer activity of zerumbone is through its inhibitory activity on tubulin polymerization and mitotic arrest.

Results from LC‐MS and NMR were in conformity with the molecular weight (218.34) and the structure of the compound. The data were in agreement with the earlier published reports.^25^ Our cell culture studies indicated that zerumbone exerted selective toxicity against HeLa cells compared to L929 cells. The half‐maximal inhibitory concentration for L929 (30 μmol/L) cells was 2‐fold higher than that for the HeLa cells (15 μmol/L). To further understand the preferential killing of HeLa cells, we made an attempt to measure the quantitative uptake of zerumbone using the fluorescently labelled compound fluorozerumbone. Although there are several reports on the anti‐cancer activity of zerumbone,^14^, ^15^ the internalization of zerumbone was not studied as it does not have a characteristic absorbance or fluorescence. Results from the internalization studies indicated that the uptake of fluorozerumbone was higher in the HeLa cells compared to the L929 cells. Thus, this property is highly favourable for zerumbone to be used as a relatively less toxic, safe and effective chemotherapeutic agent. Curcumin, the dihydroxy polyphenol from *Curcuma longa,* was also reported to induce selective toxicity in cancer cells due to preferential uptake by the cancer cells compared to normal cells.^29^ AO staining was used to analyse whether the cytotoxic effect of zerumbone was due to necrotic cell death or apoptosis. Apoptotic cells will appear brightly stained, hypercondensed and often fragmented chromatin in spherical or irregular shapes under fluorescent microscope.^52^ It was clearly evident that by the end of one cell cycle, most of the zerumbone‐treated cells underwent apoptosis since their characteristics were similar to those of the apoptotic cells.^52^ Molecules preventing metastasis are highly valuable in cancer chemotherapy as they can prevent the cancer cells spreading to other tissues. Results from the cell migration assays indicate that zerumbone strongly inhibited the migration of cancer cells at 5 and 10 μmol/L, which are much lower concentrations than its IC_50_. This result is in agreement with the previous report suggesting the anti‐metastatic property of zerumbone.^53^ Microtubules play a very important role in cell migration,^54^ and most of the potent tubulin‐targeted drugs inhibit the migration of the cell at concentrations lower than their IC_50_.^55^, ^56^

Since zerumbone showed excellent mitotic block and inhibited the migration of cancer cells, we analysed the effect of zerumbone on interphase and mitotic microtubules using immunofluorescence microscopy. In our study, we observed that zerumbone at IC_50_ induced moderate depolymerization of interphase microtubules in HeLa cells, while at 30 μmol/L (2 × IC_50_) and higher concentrations, it strongly depolymerized the interphase microtubules. Its effect on mitotic cells was more visible and dramatic as both bipolar and monopolar cells were observed depending on the concentration used. At the IC_50_, most of the mitotic cells had bipolar spindles, while at 30 μmol/L, most of the mitotic cells exhibited monopolar spindles, indicating that at the IC_50_, microtubules are the preferential target; however, at higher concentrations, it is quite possible that it might have additional targets. Our results are in agreement with previous report in which zerumbone inhibited the assembly of microtubule and induced apoptosis in PC‐3 and DU‐145 cells.^23^ But the capability of zerumbone to induce monopolar spindles was not explored earlier. The mitotic cells with monopolar spindles had condensed chromosomes in a rosette‐like configuration similar to that of the monastrol‐treated cells. Monopolar spindles are generally induced by drugs, which target the mitotic kinesins and mitotic kinases that are involved in the organization of mitotic spindle.^57^ Hence, we performed the docking analysis with tubulin, Eg5, Aurora kinase, Plk1, Nek2 and Kif2A. Our molecular docking results indicated that zerumbone has strong affinity towards Eg5 and Aurora A in addition to tubulin. It has been well documented that Eg5 and Aurora A play important role in the separation of centrosomes and inhibition of them could result in the formation monopolar mitotic cells.^58^, ^59^, ^60^ The nearly equal affinity of zerumbone towards Eg5 and Aurora A obtained by docking analysis indicated that it might target both the proteins or any one which can be confirmed only through in vitro analysis. It is important to note that zerumbone at its IC_50 _had more bipolar mitotic cells and at higher concentration such as 30 μmol/L had more of monopolar mitotic cells. The results suggest that tubulin could be the primary target of tubulin at the IC_50_, and it might target Eg5 or Aurora A or both at 30 μmol/L. At concentrations higher than 30 μmol/L, the number of mitotic cells decreased with simultaneous increase in the number of apoptotic cells. This behaviour is similar to other antimitotic drugs, which at higher concentration will activate the apoptotic pathways much earlier in the cell cycle.^32^, ^61^, ^62^

Results from the docking studies and the cell culture studies in which zerumbone induced a strong mitotic block and depolymerization of interphase microtubules motivated us to study its effect on purified tubulin isolated from goat brains using fluorescence spectroscopy. Tubulin heterodimer contains eight tryptophan residues, and interaction of small molecules will disturb the conformation of tubulin, leading to change in the intrinsic fluorescence contributed by the tryptophan residues.^38^ Zerumbone binding to tubulin with a *K*d of 4 μmol/L indicates that it has a high affinity for tubulin. The effects of microtubule targeted drugs on the polymerization of tubulin are also analysed in a cell‐free system using goat/bovine brain tubulin isolated in vitro* .* 37, 38 The results from the sedimentation assay and the light scattering assay of tubulin polymerization in the presence of glutamate are in excellent agreement with the results obtained from the cell culture studies, where zerumbone caused strong depolymerization of the interphase and mitotic microtubules in HeLa cells. Since zerumbone induced depolymerization of microtubules, and the docking analysis predicted colchicine binding domain as the binding site of zerumbone, we decided to confirm the binding site using competition binding assay using two different approaches. In one method, the change in the fluorescence of colchicine‐tubulin complex upon addition of increasing concentrations of zerumbone was monitored. In the other approach, the change in the fluorescence of tubulin‐fluorozerumbone upon addition of different compounds was monitored. Fluorescently labelled molecules are widely used to characterize the binding interactions and binding site of the ligand on the protein.^63^ Unlabelled zerumbone effectively quenched the fluorescence of the T‐C complex; similarly, colchicine could quench the fluorescence of fluorozerumbone through competitive displacement, indicating that fluorozerumbone, zerumbone and colchicine have overlapping binding sites. To further confirm that the observed quenching of fluorozerumbone is not due to artefact induced by the labelled molecule, we have used EDF in the place of fluorozerumbone and found that colchicine could not quench the FITC fluorescence.

Combination therapy which uses two or more drugs is excellent for cancer treatment because it is more effective against tumour growth and metastasis. It can destruct the cancer stem cell populations and induce apoptosis in cancer cells with less chance for the development of drug resistance.^64^ Hence, we analysed the cytotoxic activity of zerumbone in combination with the two clinically established anti‐cancer drugs vinblastine and paclitaxel. Our results suggest that zerumbone inhibited the proliferation of HeLa cells in a synergistic manner in combination with both vinblastine and paclitaxel. The strong synergistic activity of zerumbone on the inhibition of cell proliferation was due to the synergistic activity of the two drugs in controlling the cell cycle at the metaphase/anaphase transition. Although the actions of zerumbone on HeLa cells were similar to those of the microtubule depolymerizing drug colchicine, it exhibited strong mitotic block when combined with a MT‐stabilizing drug such as paclitaxel or a MT‐destabilizing drug such as vinblastine at very low concentrations. It is well documented that microtubule polymerization inducing agents and microtubule depolymerizing agents inhibit the cell proliferation at the IC_50_ only by suppressing the microtubule dynamics.^34^, ^65^ It is reasonable to assume that the synergistic activity of zerumbone with vinblastine and taxol could be due to the strong suppression of microtubule dynamics.

The mechanism of action of zerumbone was similar to that of the other clinically used chemotherapeutic drugs such as vinblastine and paclitaxel, which bind to tubulin and induce mitotic block. In addition to tubulin, zerumbone might have another target involved in centrosome separation that gets inhibited only at higher concentrations. Our finding that internalization of zerumbone is higher in cancer cells leading to it preferential killing and that it is highly effective in preventing the migration of cancer cells will be more valuable in cancer therapy either alone or in combination with other established chemotherapeutic drugs.

## CONFLICT OF INTEREST

The authors declare that there are no conflict of interests.
